# Der Effekt von mobilen Luftfiltersystemen auf die Aerosolbelastung in Großraumszenarien vor dem Hintergrund des Infektionsrisikos der COVID-19-Erkrankung. Kann die Präsenzlehre wieder aufgenommen werden?

**DOI:** 10.1007/s40664-021-00435-9

**Published:** 2021-06-22

**Authors:** M. Oberst, T. Klar, A. Heinrich

**Affiliations:** 1 Klinik für Orthopädie, Unfall- und Wirbelsäulenchirurgie, Ostalb-Klinikum Aalen, Im Kälblesrain 1, 73430 Aalen, Deutschland; 2 Zentrum für Optische Technologien (ZOP), Hochschule Aalen, Beethovenstr. 1, 73430 Aalen, Deutschland

**Keywords:** SARS-CoV‑2, Coronavirus, Pandemie, Infektion, Raumluftfilter, SARS-CoV‑2, Coronavirus, Pandemic, Infection, Room air filter

## Abstract

**Hintergrund:**

Vor dem Hintergrund der Corona-Pandemie wurde mit Beginn des Sommersemesters 2020 bundesweit die studentische Präsenzlehre praktisch eingestellt. Ebenso wurden im Rahmen des zweiten Lockdowns in vielen Bundesländern Schulen und Kindertagesstätten geschlossen bzw. auf ein Minimum heruntergefahren. In diesem Zusammenhang wurde bereits mehrfach der Effekt von Raumluftfiltern diskutiert, nach Ansicht des Umweltbundesamtes ist der Einsatz von mobilen Luftreinigern derzeit allerdings nicht empfohlen. Die vorliegende Untersuchung zeigt die konkreten Auswirkungen von mobilen Raumluftfiltern auf die Aerosolbelastung in einem Hörsaal, einer Kantine und einem schulischen Lernzentrum auf.

**Methoden:**

In 3 Großräumen (studentischer Hörsaal, Betriebskantine, Lernzentrum eines Gymnasiums) wurde der Effekt von mobilen Raumluftfiltern (Fa. DEMA-airtech, Stuttgart) gemessen. Die Messungen der Aerosolkonzentrationen bzw. der CO_2_-Werte erfolgte mit Geräten der Firma Palas, Karlsruhe.

**Ergebnisse:**

In allen 3 Szenarien zeigte sich übereinstimmend, dass die Aerosolkonzentration durch die Filter relevant und dauerhaft reduziert werden konnten. Der Effekt erschien teilweise sogar nachhaltiger und effektiver als beim normalen Stoßlüften.

**Schlussfolgerung:**

Die Verwendung von Raumluftfiltern als ergänzende Maßnahme zum Abstandhalten und dem Tragen einer effektiven Mund-Nase-Bedeckung ist eine empfehlenswerte Maßnahme, die die Wiederaufnahme der Präsenzlehre ermöglichen könnte.

## Abstract (en)

**Background:**

As a consequence of the corona pandemic, universities nationwide had stopped classroom teaching by the start of the summer semester 2020. As part of the second lockdown, in many states schools and day care centers were also closed or reduced to a minimum. In this context the effect of room air filters has already been discussed multiple times; however, mobile devices for air filtration are currently not recommended by the German Federal Environment Agency. The following investigation shows the real effects of mobile air filters on aerosol concentrations when used in lecture theaters, canteens or school learning centers.

**Methods:**

The effects of a mobile air purifier (DEMA-airtech, Stuttgart, Germany) were measured in three large rooms (a lecture theater, a company canteen and a learning center of a grammar school). Aerosol and carbon dioxide concentrations were determined with devices from the company Palas (Karlsruhe, Germany).

**Results:**

All three scenarios showed a relevant and permanent decrease in aerosol concentrations through the use of air filters. The effect partly even surpassed the effectiveness of simple ventilation by opening the windows.

**Conclusion:**

In addition to social distancing and wearing highly efficient face masks, the use air filters is recommended. This measure could enable classroom teaching to be resumed.

Vor dem Hintergrund der sich dynamisch und zuletzt unkontrolliert entwickelnden Corona-Pandemie wurde mit Beginn des Sommersemester 2020 bundesweit die studentische Präsenzlehre praktisch eingestellt [32]. Ebenso wurden im Rahmen des zweiten Lockdowns in vielen Bundesländern Schulen und Kindertagesstätten geschlossen bzw. auf ein Minimum heruntergefahren [12, 13]. Hintergrund dieser in der Geschichte der Bundesrepublik bislang einzigartigen Maßnahmen war die Vorgabe, die Aerosolübertragung des SARS-CoV-2-Virus durch konsequentes Verhindern von „Face-to-Face“-Kontakten von Schülern, Studierenden und Lehrpersonal zu unterbinden.

In diesem Zusammenhang wurde bereits mehrfach der Effekt von Raumluftfiltern diskutiert: Nach Ansicht der IRK (Innenraumluft-Hygienekommission) des Umweltbundesamtes reicht der Einsatz von mobilen Luftreinigern mit integrierten HEPA(„high efficency particulate air“)-Filter in Klassenräumen allerdings nicht aus, um wirkungsvoll über die gesamte Unterrichtsdauer Schwebepartikel (Aerosolpartikel, die ggf. Viren enthalten) aus der Raumluft zu entfernen [35]. Die Deutsche Gesellschaft für Krankenhaushygiene sieht Ende vergangenen Jahres „erheblichen weiteren Forschungs- und Entwicklungsbedarf“ [14]. Vor diesem Hintergrund präsentiert die vorliegende Untersuchung die konkreten Auswirkungen von mobilen Raumluftfiltern auf die Aerosolbelastung in realen „Vor-Ort-Situationen“ in einem studentischen Hörsaal, einer Betriebskantine und in den Räumlichkeiten einer weiterführenden Schule (Gymnasium).

## Methode

**Figure 1:**
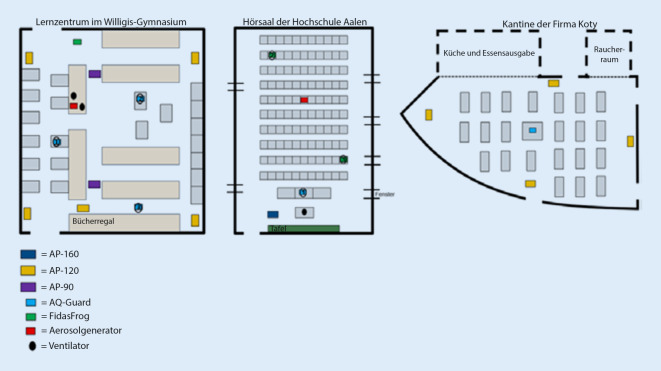

Im November und Dezember 2020 sowie im Januar 2021 wurden mobile Raumluftfilter (Firma DEMA-airtech, Stuttgart) in folgenden Großraumszenarien eingesetzt: In der Betriebskantine der Firma Coty in Köln, im Hörsaal des Zentrums für Optische Technologien (ZOP) an der Hochschule Aalen und im Lernzentrum des Willigis-Gymnasiums in Mainz. Die Messungen der Aerosolkonzentrationen bzw. der CO_2_-Werte erfolgten mit Geräten der Firma Palas, Karlsruhe. Zur Erzeugung von artifiziellen Aerosolen wurde ebenfalls ein Gerät der Firma Palas eingesetzt. Die weiteren Spezifikationen der Räume sowie die verwendeten Mess- bzw. Filtertechniken sind in Tab. 1 und Abb. 1 zusammengefasst.

**Table 1:** 

	Kantine Köln	Hörsaal Aalen	Gymnasium Mainz
Tag der Messung	10.12.2020	25.11.2020	27.01.2021
Grundfläche (m^2^)	245	114	394
Raumvolumen (m^3^)	717	296	1379
Anzahl Filtergeräte	4	1	7
CADR (m^3^/h)^a^	4800	1300	7440
Gerätetypen^b^	AP-120	AP 160	AP-120, AP-90
Messtechnik zur Aufzeichnung der Aerosolbelastung	Palas AQ-Guard	Palas Fidas Frog Palas AQ-Guard	Palas Fidas Frog Palas AQ-Guard
Messdauer (min)	140	120	250
Publikumsverkehr	Ja	Nein	Nein
Gemessene Parameter	C_N_/PM_1,0_/PM_2,5_/PM_4_/PM_10_/PM_tot_/CO_2_	C_N_/PM_1,0_/PM_2,5_/PM_4_/PM_10_/PM_tot_	C_N_/PM_1,0_/PM_2,5_/PM_4_/PM_10_/PM_tot_
Aerosolgenerator	Nein	Palas PAG 100	Palas PAG 100

*PM* Partikelmasse (Feinstaubfraktionen) in µm/m^3^ für verschiedene Größenbereiche (1,0–10 = aerodynamische Durchmesser in µm), *C*_*N*_ Gesamtpartikelanzahl in 1/cm^3^ (Messbereich von 0,18–18 µm), *CO*_*2*_ in ppm

^a^Clean Air Delivery Rate (*CADR*) = Filtereffizienz x Luftumwälzung pro Stunde anhand der eingesetzten Filter

^b^Gerätetypen/Filterkapazität: AP-90 (780 m^3^/h)/AP-120 (1200 m^3^/h)/AP-160 (1300 m^3^/h)

## Ergebnisse

### Köln (Betriebskantine Firma Coty).

**Figure 2:**
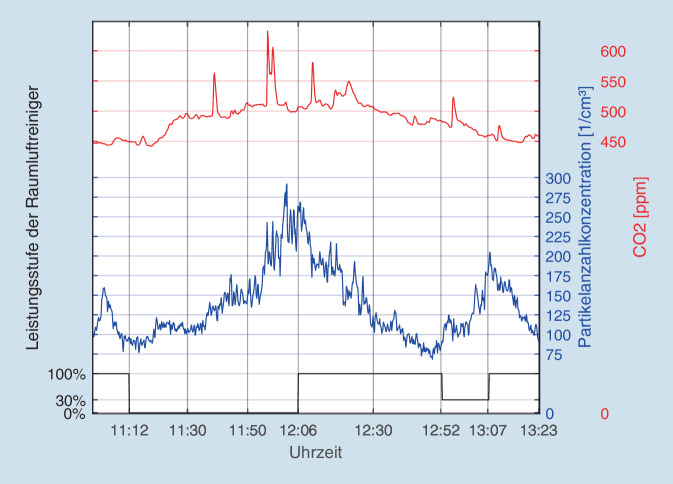

Die Abb. 2 zeigt die Partikelanzahlkonzentration C_N_ in 1/cm^3^ (AQ Guard, Messbereich 0,18–18 µm) innerhalb der 2‑stündigen „Hochphase“ der Mittagspause in der Betriebskantine der Firma Coty von 11:00 bis 13:30 Uhr. Zuvor waren die Raumluftfilter 1 h lang unter Volllast gefahren worden. Mit Beginn der mittäglichen Pausenzeit um kurz nach 11 Uhr wurden die Filter abgeschaltet. Über die nachfolgende Essenszeit zeigte sich erwartungsgemäß die höchste Besucherfrequenz vor Ort mit bis zu 50 Personen gleichzeitig im Raum. Hierunter steigt die Aerosolkonzentration deutlich an und verdreifacht sich während dieser Phase. Das Zuschalten der Luftreiniger (12:06 Uhr) reduziert – unter kontinuierlich hoher Personenfrequentierung der Kantine – die Anzahl der Gesamtteilchen auf den Ausgangswert. Die Halbwertszeit (Abfall von 250 1/cm^3^ auf 125 1/cm^3^) betrug hierbei 24 min. Die versuchsweise temporäre Reduktion der Filtergeräte auf 30 %ige Leistung (12:52 Uhr) führte dann kurzfristig wieder zu einem deutlichen Anstieg der Gesamtpartikel. Selbiger konnte durch erneutes Hochschalten der Filterleistung auf 100 % rasch wieder auf den Ausgangswert reduziert werden.

**Figure 3:**
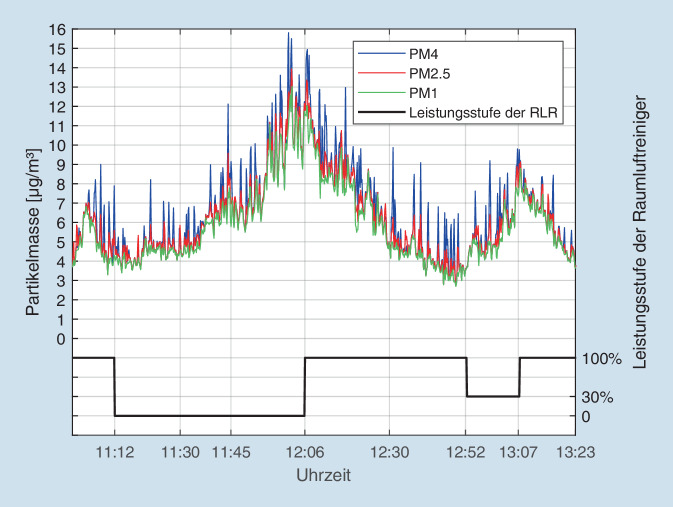

Innerhalb der ersten Stunde kam es zu einem leichten Anstieg der CO_2_-Konzentration um ca. 60 ppm (ca. 15 %), die sich zum Ende der Messung dann wieder dem Ausgangswert annäherte. Ein Effekt der veränderten Filterleistung wie bei der Aerosolkonzentration (12:52–13:07 Uhr) konnte nicht festgestellt werden. Die Abb. 3 zeigt das Verhalten der verschiedenen Massefraktionen der Aerosole für PM_1_, PM_2,5_ und PM_4_ während des oben genannten Zeitraumes. Alle 3 Kurven verlaufen parallel und praktisch identisch zur Messung der Partikelanzahlkonzentration (Abb. 2, nach Angaben der Firma Palas erfolgt die Messung der umweltbedingten Massefraktionen PM_1–10_ simultan mit der Partikelanzahl und der Partikelgrößenverteilung innerhalb des angegebenen Gesamtmessbereiches von 0,18–18 µm). Der Effekt des Zuschaltens der Raumluftfilter um 12:06 Uhr bzw. dessen temporäre Reduktion um 12:52 Uhr ist ebenso wie bei den Gesamtpartikeln (s. Abb. 2) klar erkennbar.

### Mainz (Lernzentrum Willigis-Gymnasium).

**Figure 4:**
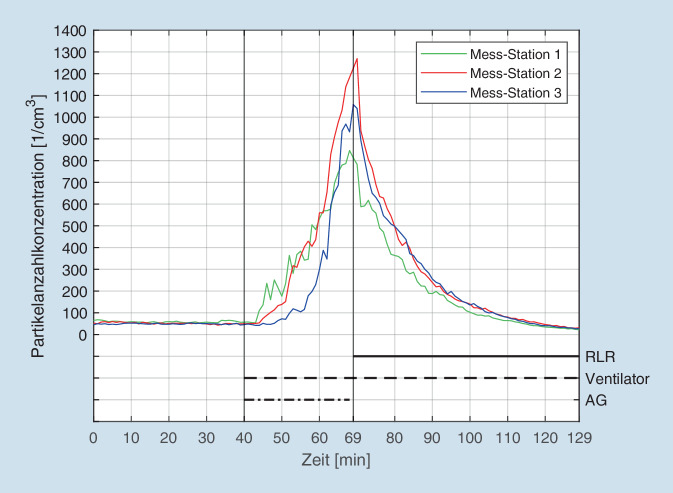

Das Lernzentrum des Willigis-Gymnasiums in Mainz war mit einem Raumvolumen von knapp 1400 m^3^ der größte der 3 untersuchten Räume. Um eine ausreichende Luftumwälzung (6-mal pro Stunde) zu erreichen, wurden insgesamt 7 Geräte im Raum verteilt (s. Tab. 1 und Abb. 1). Als Ausgangswert (Beginn der Messungen um 10:19 Uhr) i. S. d. Grundlast zeigten alle 3 Messgeräte Werte im mittleren zweistelligen Bereich (vgl. Abb. 4). Um 11 Uhr wurde zentral im Raum der Aerosolgenerator (5 % NaCl in destilliertem Wasser) gestartet, was umgehend zum Anstieg der Aerosole an allen 3 Messpunkten führte. Nach 30 min wurde der Aerosolgenerator abgeschaltet, und alle 7 Raumluftfilter wurden jeweils unter Volllast gestartet. Eine Stunde später waren an allen 3 Messpunkten die Ausgangswerte wieder erreicht. Die Halbwertszeit bis zur Reduktion der Partikellast um jeweils 50 % betrug an den 3 Messpunkten zwischen 6 min (Messpunkt 2) und 9 min (Messpunkt 1 und 3). Die jeweiligen Ausgangswerte wurden nach 42 min (Messpunkt 1) und 48 min (Messpunkt 2 und 3) erreicht. Nach weiteren ca. 14 min liegt die Partikelkonzentration an allen 3 Messpunkten bei ca. 50 % unter Ausgangsniveau.

### Aalen (Hörsaal Zentrum für Optische Technologien).

**Figure 5:**
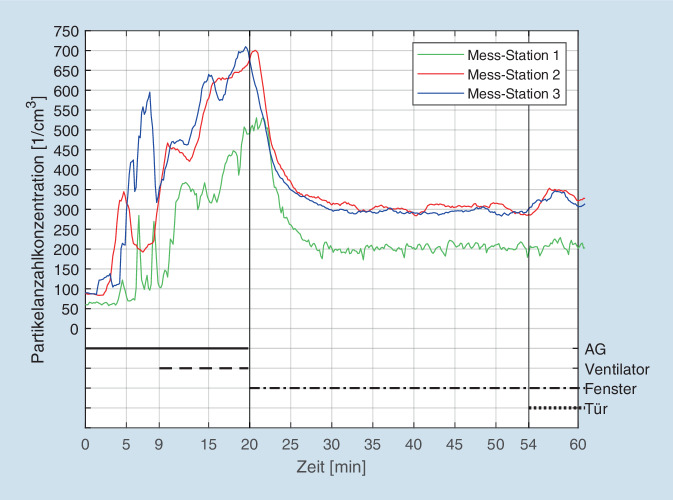

Bei den Messungen im Hörsaal des Zentrums für Optische Technologien in Aalen wurde neben dem Effekt der Raumluftfilter auch der Einfluss von „normalem Lüften“ untersucht. Die Abb. 5 zeigt den entsprechenden Verlauf der Gesamtpartikel. Zunächst steigen die Werte nach Start des Aerosolgenerators (Zeitpunkt 0) an allen 3 Messpunkten an und erreichen nach 20-minütigem Betrieb des Generators Höchstwerte von jeweils ca. 500 bzw. 700 1/cm^3^. Nach dem Öffnen der Fenster kommt es zu einem relativ raschen Abfall der Gesamtpartikel bis zu Werten zwischen 350 und 200 1/cm^3^. Eine Rückkehr zum Ausgangsniveau (50–100 1/cm^3^) wird allerdings nicht erreicht, die Werte stagnieren auf diesem Niveau trotz weiterhin geöffneter Fenster. In den letzten 6 min (zusätzliches Öffnen der Tür des Hörsaales) kommt es wieder zum Anstieg der Aerosole im Bereich der Messpunkte 2 + 3.

**Figure 6:**
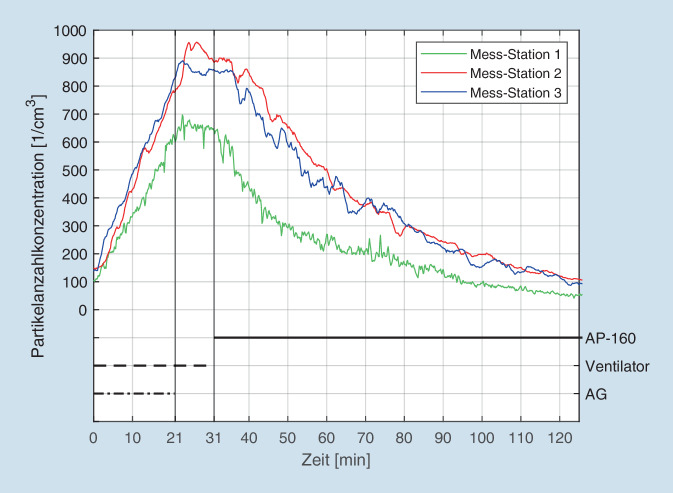

Unter den gleichen Versuchsbedingungen zeigen die Kurven in Abb. 6 den Verlauf der Gesamtpartikel unter dem Einfluss eines Luftreinigers. Der Abfall der Partikelkurve ist weniger steil als beim Lüften, dafür aber konstant abfallend. Eine Stagnation der Werte findet nicht statt. Die Aerosolkonzentration sinkt kontinuierlich und unterschreitet nach ca. 100 min sogar den ursprünglichen Ausgangswert.

## Diskussion

Bei der Übertragung von SARS-CoV‑2 von Mensch zu Mensch scheinen Virus-kontaminierte Luftschwebeteilchen (Aerosole) eine entscheidende Rolle bei der Infektion zu spielen [9, 11, 28, 31, 37]. In Abhängigkeit vom „Ausstoß“ (Sprechen, Atmen, Singen, Husten, Niesen …) werden Partikel verschiedener Größe produziert und in die Raumluft abgegeben. Hierbei kann es in geschlossenen Räumen nachgewiesenermaßen auch über mehrere Meter hinweg zur Übertragung kommen, und kontaminierte Luftpartikel oder das Virus selbst können mehrere Stunden in der Raumluft nachgewiesen werden [23, 27, 28, 36]. Besondere Bedeutung kommt vor diesem Hintergrund Aerosolen von einer Größe von < 5 µm zu, da diese kleinen und kleinsten Teilchen bereits beim normalen Atmen und Sprechen entstehen und tief in den Respirationstrakt bis auf Ebene der Alveolen vordringen können [2, 23, 26, 36].

Vor dem Hintergrund dieser Übertragungswege wurde – mangels kausaler Therapie für COVID-19 und ausstehender flächendeckender Impfung – bundesweit die Präsenzlehre sowohl in Schulen als auch Universitäten weitestgehend heruntergefahren [12, 13, 32]. Über Sinnhaftigkeit, Ausmaß und Dauer dieser Maßnahmen wird seither praktisch tagtäglich auf verschiedensten Ebenen diskutiert [3, 15, 16]. In ersten Untersuchungen konnten Schulen und Kindergärten zunächst nicht als „Pandemietreiber“ identifiziert werden [12], zwischenzeitlich mehren sich allerdings die Hinweise, dass auch Kinder und Jugendliche einen erheblichen Anteil der Übertragungen von SARS-CoV‑2 innerhalb der Gesamtbevölkerung ausmachen [10, 22]. Ergebnisse einer Langzeitkohortenstudie aus der Schweiz stehen derzeit noch aus [34].

Die Berufsgenossenschaften als Vertreter der DGUV (Deutsche Gesetzliche Unfallversicherung) haben ihren Mitgliedern bereits im Sommer 2020 die Möglichkeit der Verwendung von Raumluftfiltern aufgezeigt und Handlungsanweisungen diesbezüglich veröffentlicht [20]. Das Umweltbundesamt weist jedoch darauf hin, dass der Einsatz von mobilen Luftreinigern mit integrierten HEPA-Filtern in Klassenräumen nicht ausreiche, um wirkungsvoll über die gesamte Unterrichtsdauer Schwebepartikel (z. B. Viren) aus der Raumluft zu entfernen [35].

Demgegenüber konnte sowohl unter Laborbedingungen [5, 21] als auch in vivo [7] bereits nachgewiesen werden, dass Raumluftfiltersysteme durchaus einen relevanten Beitrag zur Reduktion der Aerosolkonzentration im Raum leisten können. In einer mathematischen Risikokalkulation der Infektion mit SARS-CoV‑2 erreichte die „high efficiency HEPA filtration“ eine 6‑ bis 8‑fache Risikoreduktion [25]. Christopherson et al. kamen aufgrund theoretischer Überlegungen zu der Überzeugung, dass mobile Luftfilter als zusätzliche Maßnahmen zum Schutz vor einer Infektion mit SARS-CoV‑2 in Betracht gezogen werden müssen [6]. In Untersuchungsräumen mit limitierten Lüftungsmöglichkeiten konnte auch unter den realen Bedingungen einer chirurgischen Sprechstunde nachgewiesen werden, dass mobile Raumluftfilter die Aerosolkonzentration relevant verringern [29, 30].

Die vorliegenden Ergebnisse konnten nun in 3 verschiedenen Großraumszenarien unter jeweils verschiedenen örtlichen Bedingungen übereinstimmend nachweisen, dass Raumluftfilter auch unter „Alltagsbedingungen“ in großen Räumen die Aerosollast konsequent und anhaltend reduzieren können. Die Anzahl der hierzu in den jeweiligen Räumen notwendigen Filter orientierte sich hierbei an der Raumgröße bzw. dem sich hieraus ergebenden Luftvolumen, welches gemäß den Vorgaben des Umweltbundesamtes 5‑ bis 6‑mal pro Stunde ausgetauscht werden soll [35]. Somit musste im relativ kleinen Hörsaal der Hochschule in Aalen nur 1 Gerät platziert werden, im großen Lernsaal des Willigis-Gymnasiums wurden hingegen 7 Filtergeräte platziert, um das große Raumvolumen von knapp 1400 m^3^ ausreichend umwälzen zu können.

Die Ergebnisse der Messungen im Hörsaal in Aalen belegen, dass die Raumluftreiniger unter bestimmten Umständen eine effektivere und dauerhaftere Reduktion der Aerosolkonzentration bewirken als einfaches Lüften: Nach Öffnen der Hörsaalfenster zeigte sich zunächst ein relativ starker Abfall der Aerosolkonzentration, der sich dadurch erklärt, dass bei großer Temperaturdifferenz zwischen Außen- und Innenluft der Luftaustausch besonders effektiv ist (Außentemperatur Aalen am 25.11.2020: −1 °C) [18]. Nach ca. 10 min erreichen die Werte dann allerdings ein Plateau bei Werten von 200 bis 300 Partikel pro cm^3^. Dieses Niveau wird trotz weiter geöffneter Fenster nicht weiter unterschritten (vgl. Abb. 5). Dies erklärt sich zwanglos durch die in der Außenluft typischerweise vorliegenden Anzahlkonzentrationen im Bereich von 300 bis 400 Partikel pro cm^3^. Der durch Lüften von außen zugeführte „natürliche Feinstaub“ unterhält das Niveau im Hörsaal auf dem festgestellten Level. Die Tatsache, dass die Aerosolkonzentration zum Ende der Messungen sogar wieder anstieg, führen wir darauf zurück, dass in unmittelbarer Nähe zur Hochschule in Aalen eine vielbefahrene Bundesstraße (B29) verläuft. Durch Öffnen nicht nur der Hörsaalfenster, sondern auch der Hörsaaltür gegen Ende der Messungen entstand offenbar ein Durchzug, der den Anteil an durchströmender Luft nochmals verstärkte. Allerdings wies diese „Frischluft“ offensichtlich eine hohe Feinstaubkonzentration auf, weswegen die Aerosolkonzentration im Raum weiter anstieg. Einschränkend muss zu dieser Interpretation allerdings gesagt werden, dass hinsichtlich des konkreten Luftaustausches beim Lüften eine Vielzahl von Parametern (Temperatur, Luftdruck, Luftfeuchtigkeit, Windgeschwindigkeit und -richtung, Sonneneinstrahlung, Reflexion etc.) einen Einfluss hat [8], deren konkrete einzelne Auswirkungen im Rahmen der vorliegenden Untersuchung nicht erfasst wurden.

Demgegenüber verlief die Reduktion der Aerosole unter Verwendung der Raumluftfilter zwar langsamer als beim Lüften (Abfallkurve weniger steil), allerdings konnten die Filtergeräte die Aerosolkonzentration im weiteren Verlauf konstant weiter absenken, zuletzt sogar bis unterhalb der Ausgangswerte (vgl. Abb. 5). Dass hierbei am Messpunkt 1 (grüne Kurve) konstant ca. 25 % niedrigere Werte als an den Messpunkten 2 und 3 festgestellt werden konnten, erklärt sich aufgrund unterschiedlicher Ausgangskalibrierung der verwendeten Messgeräte AQ-Guard/Fidas Frog.

Die Messergebnisse in der Betriebskantine in Köln sind insofern bemerkenswert, dass die Werte unter kontinuierlich laufendem Betrieb mit normalem Publikumsverkehr während der Mittagspause des Betriebes erhoben worden waren. In diesem Setup wird durch die ausgeatmete Atemluft der anwesenden Personen ständig CO_2_ produziert. Dies spiegelt sich im langsamen Anstieg der CO_2_-Konzentration ab 11:00 Uhr wider. Gegen Ende der Mittagszeit, mit Abnahme der Besucherfrequenz der Kantine, nimmt auch die CO_2_-Konzentration entsprechend wieder ab. Ein Effekt der Raumluftfilter auf die CO_2_-Konzentration war erwartungsgemäß nicht festzustellen, da die Geräte keinen Effekt auf das Luftgas CO_2_ ausüben. Auch Lee et al. konnten in einem ähnlichen Szenario (Krankenhausräume) keinen Effekt von Raumluftfiltern auf die CO_2_-Konzentration feststellen [24]. Der Effekt der Filter auf die Aerosolkonzentration bzw. -masse hingegen war eindeutig (vgl. Abb. 2 und 3).

## Limitationen

Das konkrete Risiko einer Infektion mit SARS-CoV‑2 für einzelne Personen wurde unter den genannten Bedingungen selbstverständlich nicht erfasst. Auch wenn offenbar bereits Freiwillige für „Menschenversuche“ angeworben werden (vgl. https://ukcovidchallenge.com), ist eine wissentliche Exposition mit SARS-CoV‑2 behafteten Aerosolen aus ethischer Sicht kritisch zu betrachten. Aufgrund des oben genannten bekannten Übertragungsweges der Viruserkrankung ist daher die indirekte Betrachtung der Aerosolkonzentration im Raum hinsichtlich des Risikos einer potenziellen Virusinfektion aus unserer Sicht daher nach wie vor alternativlos.

Während es auf dem Markt eine Reihe von Luftfiltern gibt, die „nur“ mit einem HEPA-Filter ausgerüstet sind, wird bei den in unseren Untersuchungen verwendeten Geräten auch ein Aktivkohlefilter eingesetzt sowie eine „Plasmadesinfektion“ und eine Titanoxydphotokatalyse durch UV-Licht Bestrahlung der durchströmenden Luft durchgeführt. Dies soll laut Herstellerangaben zusätzlich zur Filterung zu einer Abtötung von Viren und Bakterien von über 99 % führen [19]. Bei diesen Prozessen können im Rahmen der stattfindenden chemischen Reaktionen allerdings potenziell gesundheitsgefährdende Zerfallsprodukte entstehen (z. B. Ozon, Stickoxide, organische Substanzen) [1, 20, 35]. Ob und in welchem Ausmaß dies tatsächlich der Fall ist, wurde in den vorliegenden Untersuchungen nicht gemessen bzw. überprüft. Nach Angaben des Herstellers der verwendeten Raumluftfilter wurden die entsprechenden CE-Zertifizierungen bzw. Prüfprotokolle für die Geräte erbracht [17], was ein entsprechendes Maß an Sicherheit hinsichtlich der unbeabsichtigten Verbreitung von gesundheitsgefährlichen Nebenprodukten bieten sollte. Bereits 2015 wurde darauf hingewiesen, dass es bislang keine verbindlichen gesetzlichen Vorgaben für Raumluftfiltergeräte gibt [4]. Nach unserer Kenntnis existieren seitens der Verordnungsgeber allerdings nach wie vor bislang keine entsprechenden Regelungen hinsichtlich der einzuhaltenden Grenzwerte für potenzielle Abscheidestoffe von Raumluftfiltern.

## Schlussfolgerung

Die vorliegende Arbeit konnte anhand von 3 verschiedenen Großraumszenarien nachweisen, dass mobile Raumluftfilter in der Lage sind, die Aerosolkonzentration auch in großen Räumen relevant und nachhaltig zu reduzieren. Dies zeigte sich übereinstimmend in allen 3 Szenarien (vgl. Ergebnisse). Hierbei zeigte sich auch, dass die Reduktion sehr rasch erfolgt, die entsprechenden Halbwertszeiten des Abfalls der Partikelkonzentrationen der Aerosole betrugen je nach Lokalisation nur wenige Minuten bis maximal knapp eine halbe Stunde. Da sich mit der Reduktion der Aerosolkonzentration im Raum das indirekte Übertragungsrisiko einer durch viruskontaminierte Aerosole übertragenen Erkrankung wie Covid-19 äquivalent reduziert, kann geschlussfolgert werden, dass sich das Risiko für eine Infektion mit SARS-CoV-2-Viren durch mobile Raumluftfilter auch in großen Räumen relevant und effektiv reduzieren lässt. Unsere Studie bestätigt somit die theoretischen Überlegungen anderer Autoren hinsichtlich der Reduktion des Infektionsrisikos mit SARS-CoV‑2 durch Raumluftfilter [6, 25]. Ob durch die Verwendung dieser Filter sogar das Lüften unterbleiben kann, sollte vor dem Hintergrund unserer Untersuchungen diskutiert werden. Die Filter reduzieren die Aerosolkonzentration kontinuierlich. In einem geschlossenen Raum ohne Publikumsverkehr kann so theoretisch – unter Annahme einer Elimination erster Ordnung (Eliminationsgeschwindigkeit sinkt bei abnehmender Aerosolkonzentration) – nach 7 Halbwertszeiten die ursprüngliche Aerosolkonzentration um mehr als 99 % reduziert werden. Dieser Effekt wird durch das Lüften zunichtegemacht, da mit der „Frischluft“ erneut große Mengen an Aerosolen zugeführt werden. Selbige sind natürlich in aller Regel nicht mit SARS-CoV-2-Viren behaftet. In Abhängigkeit vom Standort (hier: Nähe zu einer viel befahrenen Bundesstraße) bzw. der Jahreszeit (Frühjahr) können aber andere, potenziell gesundheitsgefährdende Luftschwebeteilchen (Feinstaub, Pollen) in den Raum gelangen.

Insgesamt halten wir in Übereinstimmung mit anderen Autoren die Verwendung von Raumluftfiltern für empfehlenswert [5, 7, 21, 28–30, 33]. Besonders schulische und studentische Lehre in großen Räumen (Klassenzimmer/Hörsäle) erscheint vor diesem Hintergrund wieder denkbar, wobei die Filter selbstverständlich in ausreichender Leistungsfähigkeit (CADR entsprechend dem vorhandenen Raumvolumen) und nur ergänzend zur Abstandsregel und zum Tragen von hochwertigem Mund-Nasen-Schutz zum Einsatz gebracht werden können. Einschränkend muss darauf hingewiesen werden, dass die möglichen Abscheideprodukte der Filter, die bei der Plasmafilterung- bzw. UV-C-Bestrahlung des Luftstromes potenziell entstehen können, im Rahmen der vorliegenden Untersuchung nicht gemessen wurden. Selbiges muss in weiteren Untersuchungen überprüft bzw. künftig vom Verordnungsgeber durch entsprechende Normen definiert werden.
